# Genetics in Behcet’s Disease: An Update Review

**DOI:** 10.3389/fopht.2022.916887

**Published:** 2022-06-03

**Authors:** Yu Gao, Zhenyu Zhong, Peizeng Yang

**Affiliations:** The First Affiliated Hospital of Chongqing Medical University, Chongqing Key Laboratory of Ophthalmology, Chongqing Eye Institute, and Chongqing Branch of National Clinical Research Center for Ocular Diseases, Chongqing, China

**Keywords:** Behcet’s disease, genetics, single nucleotide polymorphism, copy number variation, epigenetic modification

## Abstract

Behcet’s disease (BD) is one of the most vision-threatening clinical entities of uveitis. Although the etiopathogenesis of BD remains obscure, accumulating evidence has demonstrated that both genetic and environmental factors may contribute to the development of BD. Genome-wide association studies (GWAS) and candidate association studies have identified several genetic variants strongly associated with BD, including variants in human leukocyte antigen (HLA) -A02, -A03, -A24, -A26, -A31, -B15, -B27, -B35, -B49, -B51, -B57, -B58, -C0704, CIITA, ERAP1, MICA, IL1A-IL1B, IL10, IL12, IL23R, IL-23R/IL-12RB2, IL1RL1-IL18R1, STAT4, TFCP2L1, TRAF5, TNFAIP3, CCR1/CCR3, RIPK2, ADO-ZNF365-EGR2, KLRC4, LACC1, MEFV, IRF8, FUT2, CEBPB-PTPN1, ZMIZ1, RPS6KA4, IL10RA, SIPA1-FIBP-FOSL1, VAMP1, JRKL/CTCN5, IFNGR1 and miRNA-146a. Epigenetic modifications are also reported to play essential roles in the development of BD, including DNA methylation and histone modification. We review here the recent advances in the genetic and epigenetic factors associated with the BD pathogenesis.

## Introduction

Uveitis is a group of inflammatory ocular diseases affecting the uveal tract, retina, and retinal blood vessels. It constitutes a number of visual impairments worldwide. Behcet’s disease (BD) is one of the most vision-threatening entities of uveitis. BD patients account for 16.5% of the total uveitis patients according to a Chinese study (1). BD is an autoinflammatory disorder that affects multiple systems, characterized by intraocular inflammation, arthritis, oral and genital ulcers and skin damages (2). While young adults aged 20-40 years are the commonest age group affected by BD, it still can be seen in children and older patients (3). Young male patients are reported to be affected more frequently and more severely than female patients (4). BD affects millions of people worldwide but mainly occurs along the ‘silk road’, with a higher incidence in Turkey, China, Japan and Iran, and a lower incidence in Europe and North America (5).

Approximately 70% of BD patients suffer from ocular involvement and the most common ocular finding is uveitis (6). BD patients with uveitis usually display recurrent and refractory ocular involvement, leading to severe visual impairments even blindness. However, the pathogenesis of BD is not entirely understood. Currently, genetic and environmental factors are considered to be responsible for the development of BD. A previous study comprising 21,940,795 individuals in 12 million families in Korea demonstrated that the familial incidence and risk of BD increased a lot among first degree relatives, especially in individuals with an affected twin (165-fold) (7). As a growing number of studies focus on the genetic predisposition for BD, multiple genetic variants associated with BD have been identified (8). Particularly, susceptibility gene for BD can be divided into human leucocyte antigen (HLA) and non-HLA. Identified genetic variants for BD include single nucleotide polymorphisms (SNPs) (**Table 1**) and copy number variations (CNVs) (**Table 2**), with variants ranging from single base to large fragment changes. Epigenetic modifications are also reported to be important in the development of BD, including DNA methylation and histone modification (62). The present review aims to summarize the latest genetic and epigenetic progress on BD.

**Table 1:** Summary HLA types and non-HLA SNPs associated with Behcet’s disease.

Year	Gene	SNP	P value	Risk Allele/Genotype	OR	non-risk Allele/Genotype	Method	Reference
2009	LOC100129342	rs11206377	3.00E-04	G	1.84	A	GWAS	(9)
	KIAA1529	rs2061634	4.20E-05	G	2.04	A		
	CPVL	rs317711	1.00E-04	C	2.26	T		
	UBASH3B	rs4936742	1.50E-03	T	1.71	C		
	UBAC2	rs9513584	5.80E-03	G	1.61	A		
2009	UBAC2	rs9517668	3.61E-05	T	2.62	C	Candidate	(10)
		rs9554581	8.53E-05	T	2.48	C		
		rs9517644	1.30E-03	T	1.72	C		
		rs11069357	2.00E-03	A	1.68	G		
		rs984477	4.30E-03	G	1.65	A		
		rs9513584	1.70E-03	G	1.74	A		
		rs9554573	1.20E-03	A	1.73	G		
		rs6491493	1.10E-03	G	1.74	A		
		rs7999348	5.80E-04	G	1.78	A		
		rs17575643	1.80E-04	T	2.91	C		
		rs727263	1.00E-04	A	2.45	G		
		rs7332161	1.10E-04	A	2.43	G		
		rs912130	7.10E-03	G	1.58	A		
		rs2892976	2.30E-04	G	1.96	A		
2010	IL10	rs1518111	1.88E-08	A	1.41	G	GWAS	(11)
	IL23R-IL12RB2	rs924080	5.35E-06	A	1.31	G		
	CPLX1	rs11248047	1.26E-07	G	1.36	A		
2010	IL23R-IL12RB2	rs1495965	1.90E-11	G	1.35	A	GWAS	(12)
	IL10	rs1800872	2.10E-14	A	1.45	G		
		rs1800871	1.00E-14	T	1.45	C		
2010	STAT4	rs7574865	9.00E-03	GG	1.61	TT/GT	Candidate	(13)
2010	IL23R	rs17375018	8.88E-04	G	1.57	A	Candidate	(14)
			3.47E-04	GG	1.86	AA/AG		
		rs11209032	1.26E-03	A	1.48	G		
			2.40E-02	AA	1.69	AG/GG		
2010	HLA	HLA-A2601	5.29E-03	–	3.56	–		(15)
2010	MICA	rs2523467	2.30E-09	A	0.59	G	GWAS	(16)
		rs3094584	4.70E-10	T	1.69	C		
2011	FCRL3	rs11264799	4.40E-02	C	1.43	A	Candidate	(17)
2012	STAT3	rs2293152	2.10E-02	GG	1.71	CC/CG	Candidate	(18)
2012	TGFBR3	rs1805110	3.00E-02	CC	0.62	CT/TT	Candidate	(19)
2012	UBAC2	rs3825427	6.90E-06	T	1.50	C	Candidate	(20)
		rs9517668	3.30E-04	T	1.40	C		
		rs9517701	2.90E-05	G	1.40	A		
2012	IL10	rs1518111	2.53E-02	T	1.20	C	Candidate	(21)
	IL23R–IL12RB2	rs17375018	1.93E-06	G	1.51	A		
		rs7517847	1.23E-06	T	1.48	C		
		rs924080	1.78E-05	T	1.29	C		
		rs10489629	7.00E-04	–	1.30	–		
		rs1343151	2.00E-04	–	1.39	–		
		rs1495965	1.00E-04	–	1.33	–		
2012	STAT4	rs7574070	3.36E-07	T	1.40	C	GWAS	(22)
		rs7572482	1.30E-08	T	1.44	C		
		rs897200	6.20E-09	A	1.45	G		
	TFCP2L1	rs17006292	5.16E-09	G	4.17	A		
2012	CD40	rs4810485	6.00E-03	TT	1.98	GG/GT	Candidate	(23)
		rs1883832	1.20E-02	TT	1.73	CC/CT		
2012	CCR1/CCR3	rs13084057	1.71E–7	A	3.13	G	Candidate	(24)
		rs13092160	6.50E-08	T	3.57	C		
		rs13075270	2.76E-07	T	3.13	C		
2012	PDGFRL	rs17633132	5.20E-03	C	2.33	T	Candidate	(25)
2013	TNFAIP3	rs9494885	8.35E-10	C	1.81	T	Candidate	(26)
			1.83E-10	TC	2.03	CC/TT		
		rs10499194	5.00E-03	C	1.92	T		
			1.50E-02	CC	1.96	TC		
		rs7753873	2.50E-02	C	1.39	A		
			1.50E-02	AC	1.49	AA		
2013	CCR1-CCR3	rs7616215	4.30E-13	C	1.39	T	GWAS	(27)
	STAT4	rs7574070	1.29E-09	A	1.27	C		
	KLRC4	rs2617170	1.34E-09	T	1.28	C		
	IL12A	rs17810546	6.01E-07	A	1.55	G		
	ERAP1	rs17482078	4.73E-11	TT	4.56	CC/CT		
2013	MEFV	rs61752717	1.79E-12	M694V	2.65	–	Candidate	(28)
2013	IL23R	rs17375018	1.00E-02	G	1.37	A	Candidate	(29)
		rs7517847	1.00E-04	T	1.59	G		
		rs1343151	1.00E-02	G	1.36	A		
	IL10	rs2222202	1.00E-02	G	1.35	A		
2014	IL10	rs1800871	2.40E-02	T	1.30	C	Candidate	(30)
		rs1518111	2.60E-02	A	1.30	G		
2014	TRAF5	rs12569232	1.30E-03	G	1.48	C	Candidate	(31)
		rs6540679	1.16E-10	A	1.59	G		
	TRAFIP2	rs13210247	2.07E-07	G	2.40	A		
2015	IFI16	rs7532207	4.70E-02	T	1.41	C	Candidate	(32)
		rs6940	4.00E-02	T	1.42	C		
	AIM2	rs855873	3.10E-02	G	1.52	A		
2015	CCR1	rs7616215	5.05E-09	T	1.52	C	Candidate	(33)
	KLRC4	rs2617170	7.98E-07	C	1.41	T		
	IL12A-AS1	rs17810546	5.96E-06	G	1.92	A		
	STAT4	rs7574070	1.28E-03	A	1.24	G		
	ERAP1	rs10050860	6.96E-03	T	1.32	C		
		rs13154629	7.55E-03	T	1.32	C		
2015	MICA	MICA-A5	8.00E-03	–	–	–	Candidate	(34)
		MICA-A5.1	1.50E-02	–	–	–		
		MICA-A6	1.58E-13	–	2.08	–		
	HLA	HLA-B*51	6.21E-14	–	3.38	–		
	HLA-F-AS1	rs114854070	1.45E-04	T	1.67	C		
	PSORS1C1	rs12525170	2.82E-18	A	3.20	G		
	HLA-B-MICA	rs76546355	4.79E-34	A	4.05	G		
2015	IL12	rs17810456	9.31E-06	G	2.06	A	GWAS	(35)
2015	–	rs7528842	8.62E-04	G	1.35	A	GWAS	(36)
	FUT2	rs632111	2.09E-05	G	1.32	A		
2016	HLA	HLA-A26	4.10E-02	–	3.52	–	Candidate	(37)
		HLA-A31	5.00E-03	–	7.17	–		
		HLA-B51	1.00E-04	–	3.63	–		
2016	TNFSF4	rs1234315	5.91E-03	T	1.48	C	Candidate	(38)
2016	IL-37	rs3811047	1.60E-09	G	1.58	A	Candidate	(39)
	IL-18RAP	rs2058660	1.24E-07	G	1.33	A		
2016	CIITA	rs12932187	6.00E-07	G	1.58	C	Candidate	(40)
	NOD1	rs2075818	6.81E-05	C	1.40	G		
2016	IL12	rs1874886	3.29E-10	A	1.66	G	Candidate	(41)
	JRKL/CNTN5	rs2848479	1.62E-08	A	1.61	G		
	HLA	HLA-B51	6.82E-32	–	3.82	–		
2016	IL23R-IL12RB2	rs924080	2.52E-04	T	1.58	C	Candidate	(42)
		rs11209032	3.46E-04	A	1.45	G		
2017	IL10	rs1800871	3.45E-09	T	1.30	C	Candidate	(43)
		rs3024490	3.98E-07	T	1.32	C		
	IL23R-IL12RB2	rs924080	4.06E-08	T	1.40	C		
		rs12141431	4.00E-09	C	1.34	T		
2017	IL1A-IL1B	rs3783550	2.12E-08	G	1.33	T	Candidate	(44)
	IRF8	rs11117433	2.73E-08	G	1.59	C		
	CEBPB-PTPN1	rs913678	1.96E-09	C	1.33	T		
	IL10	rs1518110	4.55E-09	A	1.34	C		
	CCR1	rs7616215	9.60E-11	T	1.39	C		
	IL12A	rs17753641	1.45E-09	G	1.90	A		
2017	IL23R–IL12RB2	rs4655535	3.30E-05	G	1.40	A	Candidate	(45)
		rs1495966	3.80E-05	T	1.40	C		
		rs1495965	4.90E-05	C	1.40	T		
		rs6665569	4.70E-02	T	1.40	C		
2017	TNFSF4	rs1234313	1.80E-02	A	1.40	G	Candidate	(46)
	TNFSF15	rs4246905	1.37E-05	T	1.58	C		
	TNFSF8	rs7028891	2.80E-02	G	1.36	A		
2017	CEBPB-PTPN1	rs913678	2.09E-16	C	1.31	T	Candidate	(47)
	LACC1	rs9316059	2.96E-16	A	1.40	T		
	RIPK2	rs10094579	9.21E-11	A	1.32	C		
	ADO-EGR2	rs224127	5.28E-13	A	1.27	G		
2018	FCGR3A	rs428888	7.22E–7	T	1.80	C	Candidate	(48)
			1.96E–07	CT	1.90	CC		
2020	IL1RL1–IL18R1	rs12987977	2.60E-03	T	1.29	G	Candidate	(49)
			8.93E-07	GT/TT	–	GG		
		rs12999364	1.13E-02	C	1.26	T		
			3.15E-04	CC/CT	–	TT		
		rs4851569	9.72E-03	C	1.26	A		
			3.29E-04	CC/CA	–	AA		
2020	IL-27	rs153109	2.00E-02	G	–	A	Candidate	(50)
2020	TNFα	rs1800629	4.24E-02	GA	2.29	GG	Candidate	(51)
2021	HLA	HLA-B51	#######	–	5.86	–	GWAS	(52)
		HLA-A26	9.77E-18	–	2.44	–		
		HLA-C0704	6.07E-17	–	3.78	–		
	IL23R - IL12RB2	rs11209032	1.50E-13	A	1.45	G		
		rs34426521	3.24E-13	G	1.44	A		
		rs12119179	1.83E-12	C	1.43	A		
		rs1495965	5.36E-13	G	1.44	A		
	IL10	rs3021094	5.40E-05	C	1.22	A		
	STAT4	rs7572482	8.90E-06	A	1.25	G		
	ERAP1	rs1065407	8.93E-08	C	1.62	A		
		rs10050860	3.38E-05	A	1.50	G		
		rs2287987	1.66E-05	G	1.52	A		
		rs2013717	2.29E-05	C	1.51	A		
	IFNGR1	rs9376268	4.41E-11	G	1.41	A		
	LACC1	rs3764147	6.71E-14	A	1.54	G		
		rs1373904	1.60E-13	A	1.53	G		
	CEBPB - PTPN1	rs913678	1.72E-05	G	1.27	A		
	RHOH	rs9683756	2.81E-06	A	1.30	G		
	PRDM1	rs13437093	1.14E-08	A	1.33	G		
	MTHFD1L	rs12216229	5.99E-10	A	1.85	G		
	KLF4	rs10733565	8.29E-12	G	1.41	A		
		rs7018799	2.76E-11	G	1.40	A		
		rs10979075	1.36E-12	A	1.43	G		
	ZMIZ1	rs1250569	9.77E-11	A	1.39	G		
	RPS6KA4	rs6591843	9.66E-13	G	1.45	A		
		rs7130280	1.07E-14	G	1.47	A		
	SIPA1-FIBP-FOSL1	rs2448490	5.12E-07	G	1.37	A		
		rs568617	1.10E-05	G	1.25	A		
		rs10791830	1.44E-05	G	1.25	A		
	IL10RA	rs2228054	1.00E-07	G	1.37	A		
		rs2228055	8.81E-08	A	1.37	G		
	VAMP1	rs1034969	4.72E-06	C	1.28	A		
	AGBL1	rs896615	1.91E-05	A	1.27	G		
	CMIP	rs9888833	3.49E-06	A	1.32	G		
	CDH15	rs79831785	5.83E-14	A	2.13	G		
	RNA5SP459-TCF4	rs7237392	4.07E-05	G	1.25	A		
	MRPL39-JAM2	rs2829839	5.62E-05	C	1.27	A		
	GART	rs6517178	6.53E-06	G	1.27	A		
	MIS18A	rs4817467	4.04E-08	A	1.35	G		
2021	IL-35	rs428253	3.40E-02	G	5.00	C	Candidate	(53)
			2.10E-02	GG/GC	–	CC		
2021	IFNGR1	rs4896243	2.42E-09	C	1.25	T	Candidate	(54)
	LNCAROD/DKK1	rs1660760	2.75E-08	C	1.28	T		
2021	miR-146a	rs2910164	2.20E-02	G	1.58	C	Candidate	(55)
	UTS2	rs228648	3.20E-02	Thr	1.60	Met		

GWAS, Genome-wide association studies; OR, Odds ratio; SNP, Single nucleotide polymorphism.

**Table 2:** Summary CNVs associated with Behcet’s disease.

Year	Gene	CNV	P value	OR	Reference
2014	C4	≤2	1.42E-03	–	(56)
		≤4	3.56E-04	–	
2015	TLR7	>2	2.00E-02	–	(57)
2015	C3	>2	2.60E-04	3.4	(58)
	C5	>2	1.50E-05	7.7	
2015	IL17F	>2	4.17E-08	2.2	(59)
	IL23A	>2	2.86E-11	2.8	
2015	Rorc	>2	8.99E-08	3.0	(60)
	Foxp3	<2	1.92E-05	3.1	
2015	FAS	>2	3.35E-08	2.16	(61)

## Variants in Human Leucocyte Antigen and Its Related Genes

HLA is located on the chromosome 6p21.3 and functions to encode various important proteins of immune responses. HLA antigen plays a critical role in self/nonself-discrimination by presenting antigens to T cells and eliminating pathogenic microorganisms such as bacteria and viruses (63). Multiple HLA alleles have been identified to be associated with various autoimmune diseases, especially BD in different populations. HLA-A02, -A24, -A26, -A31, -B27, -B51, -B57 were considered to be BD-risk alleles, while HLA-A03, -B15, -B35, -B49, -B58 were thought to be BD-protective (15, 37, 64, 65). HLA-B51 has been recognized as the most significant susceptibility gene for BD in a large number of GWAS and candidate association studies, and it was carried in about 60% of BD patients (22, 41, 66, 67). Moreover, HLA-B51 carriers were more prone to developing ocular manifestations of BD, which was observed towards the east along the Silk Road in Eurasia rather than west-Eurasian (68). However, HLA-B51 was only responsible for 19% of the genetic susceptibility to BD (69). Recently, we discovered HLA-C0704 to be associated with BD in a GWAS study involving a Chinese Han population, including eight independent SNPs at the genome-wide level (52).

Meanwhile, multiple HLA-related gene variants also play essential roles in BD, including variants in the class II major histocompatibility complex transactivator (CIITA), endoplasmic Reticulum Aminopeptidase 1 (ERAP1) and the major histocompatibility complex class I chain related gene A (MICA) (64). CIITA is an important transcriptional coactivator that modulates expression of MHC class II genes, IL-4 and IL-10 (70). In a Chinese Han population, the CIITA SNP rs12932187 G allele and GG genotype were identified to be involved in BD (40). The expression of CIITA was increased while that of IL-10 was decreased in peripheral blood monocytes (PBMC) stimulated by Lipopolysaccharide (LPS) of GG carriers (40).

ERAP1 is an important enzyme that trims peptides for binding onto MHC class I molecules. SNP rs17482078 in ERAP1 was found as a risk factor for BD in a Turkish GWAS and the finding was replicated in Iranian (27, 33). Moreover, SNP rs10050860, rs1065407, rs2287987 and rs2013717 in ERAP1 were also associated with BD in a Chinese GWAS study (52).

MICA is a highly polymorphic non-classic HLA gene that modulates immune responses by binding to its receptors on natural killer (NK) cells, CD8 T cells, and γδT cells (63, 71, 72). The DNA sequence of MICA is highly polymorphic. A tri-nucleotide microsatellite polymorphism (GCT/AGC) n was identified in the MICA transmembrane region designated as An (A4, A5, A5.1, A6, A9) allele (63). Among them, MICA-A6 conferred risk to BD while the rest alleles were thought to be protective, especially in the Middle East and East Asia (71, 73). MICA*009 and MICA* 019 were identified as risk alleles for BD involving a Spanish population (71, 74). However, a recent study in a Han Chinese population showed that no significant difference of MICA*009 polymorphisms were observed between the BD patients and controls, which indicated genetic heterogeneity of MICA gene polymorphisms in different populations (75). Additionally, MICA*049 was firstly reported to have an association with BD in this study, and this association was suggested to be independent from that with HLA-B51. The MICA*009 allele was probably mixed with the MICA*049 allele previously, since the only difference between the MICA*00901(a subtype of MICA*009) and the MICA*049 existed at codon 335 in exon 6 which was not studied. The MICA*009 was distinguished from the MICA*049 by T-ARMS-PCR in this study (75). Consistent with previous studies, the MICA*A6 was strongly associated with BD Chinese population.

## Single Nucleotide Polymorphisms in Interleukin Family Genes

Interleukin-10 (IL-10) is a widely expressed cytokine that contributes to preventing inflammatory and autoimmune pathologies. Multiple cells of innate and adaptive immune system exert important functions in BD, including CD4^+^T cells (76, 77), CD8^+^ T cells (78), dendritic cells (79), macrophages (80), NK cells (81), neutrophils (82) and B cells (83, 84), and almost all of them could express IL-10 (85). IL-10 was identified as risk locus for many inflammatory diseases, including bacterial sepsis (86), type I diabetes (87), mixed connective tissue disease (MCTD) (88), ankylosing spondylitis (AS) (89) and especially BD (9, 11, 12, 90).

Multiple SNPs of IL-10 were confirmed to be strongly associated with BD. Residing in the intron 3 of IL-10, the SNP rs1554286 was highly associated with BD in Japanese populations (12). A meta-analysis of IL-10 with additional studies in Turkish and Korean cohorts identified that IL-10 locus rs1800872 (P=2.1×10^−14^) and rs1800871 (P=1.0×10^−14^) had genome-wide significant associations with BD (12). Association between rs1518111 polymorphism of IL-10 and risk of BD was identified by multiple studies in various populations (11, 27, 30, 34). A recent GWAS study of Chinese BD uveitis involving 978 patients and, 4388 controls also confirmed the risk factor role of the IL-10 variant (rs3021094) (52). In addition, two novel loci of IL10RA (rs2228054 and rs2228055) showed genome-wide significant association with BD uveitis in this study.

Recently, some studies demonstrated how GWAS-identified IL-10 loci contributed to BD progression. IL-10 locus rs1518111 was associated with reduced IL-10 expression, which was a risk factor for BD (11, 12). A previous study proposed that IL-10 loci polymorphism was associated with impaired M2 macrophage (anti-inflammatory) function while promoting M1 macrophage (proinflammatory)-mediated inflammation in BD (91). Our study has identified rs3024490 and rs1800871 of IL-10 as susceptibility loci for BD in Chinese patients. PBMCs of rs3024490/TT genotype carriers showed decreased IL-10 production as compared with GG carriers (43). Interestingly, we further found that the risk allele T of rs3024490, which was located in the enhancer elements of IL-10, may be involved in BD pathogenesis by affecting the binding of TBX1 to IL-10. Since decreased TBX1 expression was shown in BD patients, the binding of TBX1 to IL10 decreased and the expression of IL10 was downregulated. Therefore, the risk of developing BD increased (92). However, whether the genetic changes of TBX1 could directly influence BD remains unknown.

IL-12 is a proinflammatory cytokine which contributes to Th1 activation, NK-cell cytotoxicity and IFNγ production. IL-12 is composed of p40 subunit and p35 subunit. IL-23 is a cytokine that consists of p19 subunit and the common p40 subunit with IL-12. IL-23 functions to influence inflammatory macrophage and memory T cell *via* binding to IL-23 receptor (IL-23R). IL-12 and IL-23 were reported to be involved in autoimmune inflammations (93). A multi-center GWAS study carried out in Western Europeans, Middle Eastern and Turkish, reported the association between IL-12A/rs17810546 with BD (35). Such a finding was replicated in Iranian BD cohorts (33). The locus rs1874886 of IL-12 was found to be a BD susceptibility factor in Spaniards (41). Activation of IL-23/IL-17 pathway is important in many autoinflammatory diseases, including BD (77). IL-12Rβ1 encodes the common subunit of IL23R and IL-12R, while other subunits are encoded by IL23R and IL12RB2 respectively. Stimulated by IL-23 and IL-12, IL-23R and the IL-12RB play important roles in naïve CD4^+^T cell differentiation and promote the expression of IL-1β, IL-6, IL-17A and TNFα (94). Multiple SNPs (rs12119179, rs4655535, rs924080, rs11209032, rs1343151, rs1495965, rs1495966, rs17375018, rs7517847, rs34426521, 10489629 and rs1966176) located in IL-23R/IL-12RB2 genes were involved in BD in the populations of Turkey (11), Japanese (12), Han Chinese (14, 42, 43), Iranian (21), Korean (45), Spain (29), and Western Algeria (95). However, little is known concerning the specific molecular mechanisms how these gene variants affect the pathogenesis of BD. The specific roles of these identified SNPs are warranted to be explored in further studies.

IL1RL1–IL18R1 region contains a list of genes involved in immune responses, including IL1RL2, IL1RL1, IL18R1, and IL18RAP. These genes contribute to T cells differentiation and cytokine production, such as TNF-α, IL-17A and IL-23 (96–98). Recent studies showed that IL1RL1–IL18R1 region was involved in various immune-mediated diseases, such as asthma, atopic dermatitis and BD (99, 100). Genetic ablation of IL18R1 or blockade of IL-18 receptor signaling could protect mice from autoimmune disease (98, 101).Three SNPs (rs12999364, rs12987977, and rs4851569) located in the IL1RL1–IL18R1 region were associated with ocular manifestations in Chinese BD patients. Among them, rs12987977 showed the strongest association with BD (P value=8.93e10−7, OR=0.39). Further investigations demonstrated that rs12987977/GG genotype resulted in decreased production of IFN-γ or TNF-α, indicating its anti-inflammatory role (49).

## Single Nucleotide Polymorphisms in MicroRNA

MicroRNA (MiRNA), a member of noncoding RNAs, functions in regulating gene expression by inducing cleavage, degradation, or block translation of messenger RNA. Over the last decade, multiple studies showed that miRNAs participate in the post-transcriptional gene regulation of BD and could be used as diagnostic biomarkers (102, 103). In an Iranian population, a significantly increased expression of miR-155 was observed in the PBMCs of BD patients as compared with controls (104). Jadideslam et al. confirmed that in BD patients from Iran the production of miR-21 and miR-146b decreased significantly, while the production of miR-326 increased significantly. MiR-326 expression level was proposed to be a biomarker to predict severe ocular involvement in BD patients (105). In addition, higher proportions of the G/G genotype and G allele of miR-146a rs2910164 variant were found in BD patients as compared with the healthy (55). Other altered expression of miR-25, miR-106b, miR-326 and miR-93, miR-182, miR-638, and miR-4488 has also been described in BD (106–109). However, the specific mechanism how the variants in MiRNA confer risk to BD remains unknown.

## Single Nucleotide Polymorphisms in Other Genes

The Janus kinase (JAK)/signal transducers and activators of transcription (STAT) pathway exerts important functions in multiple immune-mediated diseases. Many clinical trials made progress in treating autoimmune diseases using JAK inhibitors, including rheumatoid arthritis (110), psoriatic arthritis (111), ulcerative colitis (112) and psoriasis (113). Several case reports also indicated that targeting JAK/STAT pathway could alleviate the progression of uveitis (114–116). Tofacitinib, a JAK1/3 inhibitor targeting T cell signaling, was shown to be effective for BD patients (117). Our previous study demonstrated that multiple SNPs of JAK1 contributed to the genetic susceptibility of BD with ocular involvement, including rs2780815, rs310241, rs3790532 (118). STAT4 belongs to the STAT family that regulate s gene transcription in response to type I interferon (IFN-I) and various cytokines of IL family (119). STAT4 has been implicated in T-helper cell differentiation, natural killer (NK) cell activation and IFNγ production and contributes to multiple inflammation and autoimmune diseases (119, 120). Recent studies have identified many susceptibility loci of STAT4 in a variety of diseases including rheumatoid arthritis (RA) (121, 122), systemic lupus erythematosus (SLE) (121, 122) and BD (13). A GWAS study initially reported susceptibility locus of rs7574070 STAT4 for BD in Turkish populations, which was replicated in Japanese, Chinese and Iranian populations (22, 27, 33). We found that SNPs rs7572482 and rs897200 in STAT4 were also shown to be associated with BD in a Han Chinese GWAS (22). Among them, the rs897200 risk genotype AA was associated with higher expression of STAT4 and severer clinical symptoms including. This GWAS in Han Chinese population totally identified 10 non-HLA SNPs which showed genome-wide significant associations with BD, including rs17006292 in TFCP2L1 for the first time (22).

TRAF5, a polymorphic gene, is a closely related member of tumor necrosis factor-receptor-associated factors (TRAF) family. It was identified to be associated with various autoimmune diseases including inflammatory bowel disease, diabetes and rheumatoid arthritis (31, 123). In, 2013, a case-control study in Han Chinese descent found that SNP rs12569232, rs10863888 and rs6540679 in TRAF5 conferred susceptibility to BD. Additionally, further investigations showed that TRAF5 gene polymorphisms contributed to regulating TRAF5 production as well as downstream inflammatory cytokines including TNF-α and IL-6 (31). Recently, some SNPs were shown to regulate the expression of long non-coding RNAs (lincRNAs) and then influence disease progression. The SNP rs12569232–residing in the non-coding site of the intergenic region between linc00467 and TRAF5 may affect the pathogenesis of BD through increasing the expression of linc00467. Moreover, the study suggested that overexpression of linc00467 enhanced cell viability of CD4^+^T cells, which was essential to immune response in BD patients (124).

Recently, SNP rs9494885 in the tumor necrosis factor alpha-inducible protein 3 (TNFAIP3) was also identified to be genome-wide significantly associated with BD susceptibility in a Chinese Han population (26). However, no differences in TNFAIP3 expression was shown in different genotypes of rs9494885 (26). Therefore, SNP rs9494885 conferred risk to BD probably through an unknown mechanism rather than directly regulating TNFAIP3 expression.

With a high prevalence of BD in Turkey, studies have reported various novel susceptibility SNPs with genome-wide significance for BD in Turkey, including rs7616215 in CCR1, rs2230801, rs10094579 in RIPK2, rs224127, rs1509966 in ADO-ZNF365-EGR2, rs2617170 in KLRC4, rs2121033 in LACC1, rs61752717 in MEFV, rs7203487, rs142105922, rs11117433 in IRF8, rs681343 in FUT2, rs913678 in CEBPB-PTPN1 (27, 28, 44). Many of those were replicated by Iranian, Japanese and Han Chinese (33, 36, 47, 64).

To demonstrate genetic association between the CCR1/CCR3 gene and BD, we carried out a two-stage case control study in Chinese Han population. The combined studies showed that three SNPs (rs13092160, rs13084057, and rs13075270) conferred risk to BD. Among them, rs13092160 reached the GWAS significance threshold (P value<5e10−8) (24). Additionally, our recent GWAS study in Chinese BD confirmed 7 previously reported variants and proposed 22 novel susceptibility loci in the non-HLA region. The genome-wide significant associations of ZMIZ1, IL10RA, RPS6KA4, SIPA1-FIBP-FOSL1 and VAMP1 with BD were identified (52). Another Chinese candidate association study showed that rs9316059 in LACC1 has an association with BD at the genome-wide level of significance (47). Additionally, a Spanish study identified a novel genetic marker for BD, SNP rs2848479 in JRKL/CTCN5, with a genome-wide association (41).

Besides confirming six previously reported susceptibility variants in BD, a study of 9,444 patients and controls from seven different populations identified two novel genome-wide significant loci: rs4896243 in IFNGR1 and rs1660760 within the intergenic region of LNCAROD/DKK1 (54). Another variant in IFNGR1, rs9376268, was also shown to be involved in BD in the Chinese GWAS (52). IFNGR1 is the gene encoding the ligand-binding chain (alpha) of the interferon-gamma receptor, playing an essential role in cell immune response in BD (52). As a growing number of genetic variants have been identified with susceptibility to BD, various studies showed the correlation between genetic variants and specific manifestations of BD (**Table 3**).

**Table 3:** The correlation between genetic variants and specific manifestations of BD.

Gene	SNP	Risk Allele/Genotype	Manifestation	Population	PMID
IL-23R	rs7517847	G	genital ulcers	Turkish population	(125)
	rs11805303	G	papulopustular lesions		
	rs1004819	G	papulopustular lesions		
MVK	rs35191208	C	neurological involvements	Turkish population	(126)
ERAP1	rs30187	TT	cardiac involvements	Iranian population	(127)
	rs30187	TT	articular involvements	Iranian HLA-B*51 positive population
IL-27	rs153109	AG/GG	joint and vascular involvements	Iranian population	(128)
CD40	rs4810485	GT	skin lesions	Turkish population	(129)
CD40	rs1883832	CC	genital ulcers		
IL-10	rs1800896	A	genital ulcer	Egyptian population	(130)
		G	ocular manifestations		
IL17A	rs8193036	TT	intestinal involvements	Korean population	(131)
IL23R	rs1884444	GG/GT	intestinal involvements		
CTLA-4	rs231775	A	ocular and vasculitic manifestations	Egyptian population	(132)
JAK1	rs2780815	G	ocular involvements	Chinese Han population	(118)
	rs310241	C	ocular involvements		
	rs3790532	A	ocular involvements		
PTPN2	rs1893217	C	gastrointestinal involvements	Chinese Han population	(133)
	rs2542151	GT	gastrointestinal involvements		
MIF	rs755622	C	oral aphthae, genital ulceration, hypopyon, and arthritis involvements	Chinese Han population	(134)
	rs2096525	C	oral aphthae, genital ulceration, hypopyon, and skin lesion		
IL-10	rs1800871	T	genital ulcers, skin lesions	Chinese Han population	(135)
ERAP1	rs17482078	GA	neurological involvements	Italian population	(136)
IL23R	rs17375018	GG	neurological involvements		

## Copy Number Variations

Recently, an increasing number of studies showed that the main cause of structural variation in the genome is copy number variation (CNV). It represents large fragments of DNA sequence variation, involving insertions, duplications, and deletions of sequences (8, 137). CNV significantly contributes to human genetic diversity by influencing the gene expression. Therefore such variations have been found to be associated with autoimmune disorders including BD (56), SLE (138), RA (139) and Grave’s disease (140).

Since complement activation is involved in various immune-mediated diseases, the association of CNV in complements with BD is worthy to study. A previous study detected the copy number of C4 in 905 patients with BD, 205 patients with ankylosing spondylitis (AS) and acute anterior uveitis (AAU), and 1,238 controls by real-time PCR test. A significantly higher ratio of more than 2 copies of C4A patients were observed in BD. However, there was no significance in AS and AAU patients. Functional studies suggested that the high copy number of C4a could moderate C4a expression and promote IL-6 production, which was regarded as a risk factor for BD (56). Additionally, BD patients showed a remarkable high frequency of covering over two copies of C3 and C5 (58).

Toll-like receptors (TLRs), members of pattern-recognition receptors (PRRs), were indicated to be associated with the pathogenesis of various inflammatory or autoimmune human diseases (141, 142). In a Chinese Han population, a research unprecedent declared that the risk of BD could be increased by high copies of the TLR7 gene. TLR7 is located on the X chromosome. The frequencies of >1 copy of TLR7 in male BD patients and >2 copies in female patients were increased, suggested the difference between genders (57).

As a growing number of studies discovered apoptosis-related genes playing critical roles in various autoimmune diseases, recent research investigated whether the CNVs of these genes were associated with BD (61, 143–145). The high copy number of FAS gene, one of the apoptosis-related genes, was identified to be strongly associated with BD (61).In addition, frequencies of more than 2 copies of IL17F and IL23A were significantly increased in Chinese male BD patients as compared with controls (59). Other CNVs associated with BD include Rorc and Foxp3 gene variants (60).

## Epigenetic Modifications

Epigenetics usually refer to the heritable changes in gene expression without the DNA sequences alterations (102). It contributes to control gene expression and modulate cell development, differentiation, and activity (146). Epigenetic modifications, containing DNA methylation and histone modification, are considered to be associated with the pathogenesis of BD (62).

DNA methylation is universally considered as the main epigenetic modification of autoimmune diseases. DNA methylation is the covalent addition of methyl groups to the fifth carbon (C5) in the cytosine base, which may regulate physiological and pathological processes (102). A study found a high expression level of IL-6 gene in BD patients with decreased promoter methylation level of the IL-6 mRNA. The change in the methylation level of the IL-6 may be associated with susceptibility to BD (147). Additionally, expression level of the IL-10 gene decreased in BD patients with a high promoter methylation level. Interestingly, the high IL-10 methylation level is significantly associated with severe symptoms in BD patients (148).

An epigenome-wide association study (EWAS) showed, 4332 BD-related differentially methylated CpG sites in Han Chinese. Among them, five methylated sites (cg03546163, cg25114611, cg20228731, cg23261343, and cg14290576) in four genes (FKBP5, FLJ43663, RUNX2, and NFIL3) were significantly hypomethylated. Especially, the study discovered significant overexpression of FKBP5 mRNA with the hypomethylation at cg03546163 and cg25114611 in FKBP5 (149).

Histone modifications, including acetylation, methylation, SUMOylation, and ubiquitination, could influence the chromatin structure and gene expression (62). Sirtuin 1 (Sirt1), a histone deacetylase, which could inhibit NF-κB transcription and T-cell proliferation, promote TNF-α induced apoptosis and affect immune tolerance through regulating histone acetylation, was considered as a risk factor for BD with uveitis (150). However, future studies are needed for the full comprehension how epigenetic modifications confer susceptibility to BD.

## Conclusion

Uveitis is a sight-threatening inflammatory ocular disease, among which BD is one of the most common and complex entities. Although the pathogenesis of BD is not entirely understood, specific HLA types and gene variants in non-HLA regions have been identified to contribute to the susceptibility of BD. Specifically, SNPs as well as CNVs play critical roles in the gene variants. In addition, epigenetic modifications including DNA methylation and histone modification were also implicated in the development of BD. However, the identified gene variants still account for a small part of the pathogenesis of BD. A large number of BD-related gene variants have yet to be explored.

## Author Contributions

YG, ZZ, and PY conceived the structure of manuscript. YG drafted initial manuscript. YG and ZZ made the tables. PY and ZZ revised this manuscript. All authors contributed to the article and approved the submitted version.

## Funding

The work was supported by the Chongqing Key Laboratory of Ophthalmology (CSTC, 2008CA5003), Chongqing Outstanding Scientists Project (2019), Chongqing Chief Medical Scientist Project (2018), Chongqing Science & Technology Platform and Base Construction Program (cstc2014pt-sy10002).

## Conflict of Interest

The authors declare that the research was conducted in the absence of any commercial or financial relationships that could be construed as a potential conflict of interest.

## Publisher’s Note

All claims expressed in this article are solely those of the authors and do not necessarily represent those of their affiliated organizations, or those of the publisher, the editors and the reviewers. Any product that may be evaluated in this article, or claim that may be made by its manufacturer, is not guaranteed or endorsed by the publisher.

## References

[1] Yang P Zhang Z Zhou H Li B Huang X Gao Y. Clinical Patterns and Characteristics of Uveitis in a Tertiary Center for Uveitis in China. Curr Eye Res (2005) 30(11):943–8. doi: 10.1080/02713680500263606 16282128

[2] Sakane T Takeno M Suzuki N Inaba G. Behcet’s Disease. N Engl J Med (1999) 341(17):1284–91. doi: 10.1056/NEJM199910213411707 10528040

[3] Kone-Paut I. Behcet’s Disease in Children, an Overview. Pediatr Rheumatol Online J (2016) 14(1):10. doi: 10.1186/s12969-016-0070-z 26887984 PMC4758175

[4] Mat MC Sevim A Fresko I Tuzun Y. Behcet’s Disease as a Systemic Disease. Clin Dermatol (2014) 32(3):435–42. doi: 10.1016/j.clindermatol.2013.11.012 24767193

[5] Mahr A Maldini C. Epidemiology of Behcet’s Disease. Rev Med Interne (2014) 35(2):81–9. doi: 10.1016/j.revmed.2013.12.005 24398415

[6] Yang P. Atlas of Uveitis. PR of China: Springer Nature Singapore Pte Ltd. and People’s Medical Publishing House (2021).

[7] Ahn HS Kim HJ Kazmi SZ Kang T Jun JB Kang MJ. Familial Risk of Behcet’s Disease Among First-Degree Relatives: A Population-Based Aggregation Study in Korea. Rheumatol (Oxford) (2021) 60(6):2697–705. doi: 10.1093/rheumatology/keaa682 33241295

[8] Hou S Li N Liao X Kijlstra A Yang P. Uveitis Genetics. Exp Eye Res (2020) 190:107853. doi: 10.1016/j.exer.2019.107853 31669406

[9] Fei Y Webb R Cobb BL Direskeneli H Saruhan-Direskeneli G Sawalha AH. Identification of Novel Genetic Susceptibility Loci for Behcet’s Disease Using a Genome-Wide Association Study. Arthritis Res Ther (2009) 11(3):R66. doi: 10.1186/ar2695 19442274 PMC2714112

[10] Sawalha AH Hughes T Nadig A Yilmaz V Aksu K Keser G. A Putative Functional Variant Within the UBAC2 Gene is Associated With Increased Risk of Behcet’s Disease. Arthritis Rheumatol (2011) 63(11):3607–12. doi: 10.1002/art.30604 PMC3205238 21918955

[11] Remmers EF Cosan F Kirino Y Ombrello MJ Abaci N Satorius C. Genome-Wide Association Study Identifies Variants in the MHC Class I, IL10, and IL23R-IL12RB2 Regions Associated With Behcet’s Disease. Nat Genet (2010) 42(8):698–702. doi: 10.1038/ng.625 20622878 PMC2923807

[12] Mizuki N Meguro A Ota M Ohno S Shiota T Kawagoe T. Genome-Wide Association Studies Identify IL23R-IL12RB2 and IL10 as Behcet’s Disease Susceptibility Loci. Nat Genet (2010) 42(8):703–6. doi: 10.1038/ng.624 20622879

[13] Hu K Yang P Jiang Z Hou S Du L Li F. STAT4 Polymorphism in a Chinese Han Population With Vogt-Koyanagi-Harada Syndrome and Behcet’s Disease. Hum Immunol (2010) 71(7):723–6. doi: 10.1016/j.humimm.2010.04.007 20438790

[14] Jiang Z Yang P Hou S Du L Xie L Zhou H. IL-23R Gene Confers Susceptibility to Behcet’s Disease in a Chinese Han Population. Ann Rheum Dis (2010) 69(7):1325–8. doi: 10.1136/ard.2009.119420 20375120

[15] Kaburaki T Takamoto M Numaga J Kawashima H Araie M Ohnogi Y. Genetic Association of HLA-A*2601 With Ocular Behcet’s Disease in Japanese Patients. Clin Exp Rheumatol (2010) 28(4 Suppl 60):S39–44. 20868569

[16] Meguro A Inoko H Ota M Katsuyama Y Oka A Okada E. Genetics of Behcet Disease Inside and Outside the MHC. Ann Rheum Dis (2010) 69(4):747–54. doi: 10.1136/ard.2009.108571 19684014

[17] Li K Zhao M Hou S Du L Kijlstra A Yang P. Association Between Polymorphisms of FCRL3, a non-HLA Gene, and Behcet’s Disease in a Chinese Population With Ophthalmic Manifestations. Mol Vis (2008) 14:2136–42. PMC2592528 19050767

[18] Hu K Hou S Jiang Z Kijlstra A Yang P. JAK2 and STAT3 Polymorphisms in a Han Chinese Population With Behcet’s Disease. Invest Ophthalmol Vis Sci (2012) 53(1):538–41. doi: 10.1167/iovs.11-8440 22205606

[19] Chen Y Yang P Li F Hou S Jiang Z Shu Q. Association Analysis of TGFBR3 Gene With Vogt-Koyanagi-Harada Disease and Behcet’s Disease in the Chinese Han Population. Curr Eye Res (2012) 37(4):312–7. doi: 10.3109/02713683.2011.635398 22440163

[20] Hou S Shu Q Jiang Z Chen Y Li F Chen F. Replication Study Confirms the Association Between UBAC2 and Behcet’s Disease in Two Independent Chinese Sets of Patients and Controls. Arthritis Res Ther (2012) 14(2):R70. doi: 10.1186/ar3789 22455605 PMC3446441

[21] Xavier JM Shahram F Davatchi F Rosa A Crespo J Abdollahi BS. Association Study of IL10 and IL23R-IL12RB2 in Iranian Patients With Behcet’s Disease. Arthritis Rheumatol (2012) 64(8):2761–72. doi: 10.1002/art.34437 22378604

[22] Hou S Yang Z Du L Jiang Z Shu Q Chen Y. Identification of a Susceptibility Locus in STAT4 for Behcet’s Disease in Han Chinese in a Genome-Wide Association Study. Arthritis Rheumatol (2012) 64(12):4104–13. doi: 10.1002/art.37708 23001997

[23] Chen F Hou S Jiang Z Chen Y Kijlstra A Rosenbaum JT. CD40 Gene Polymorphisms Confer Risk of Behcet’s Disease But Not of Vogt-Koyanagi-Harada Syndrome in a Han Chinese Population. Rheumatol (Oxford). (2012) 51(1):47–51. doi: 10.1093/rheumatology/ker345 22087016

[24] Hou S Xiao X Li F Jiang Z Kijlstra A Yang P. Two-Stage Association Study in Chinese Han Identifies Two Independent Associations in CCR1/CCR3 Locus as Candidate for Behcet’s Disease Susceptibility. Hum Genet (2012) 131(12):1841–50. doi: 10.1007/s00439-012-1200-4 22829007

[25] Hou S Xiao X Zhou Y Zhu X Li F Kijlstra A. Genetic Variant on PDGFRL Associated With Behcet Disease in Chinese Han Populations. Hum Mutat (2013) 34(1):74–8. doi: 10.1002/humu.22208 22926996

[26] Li H Liu Q Hou S Du L Zhou Q Zhou Y. TNFAIP3 Gene Polymorphisms Confer Risk for Behcet’s Disease in a Chinese Han Population. Hum Genet (2013) 132(3):293–300. doi: 10.1007/s00439-012-1250-7 23161053

[27] Kirino Y Bertsias G Ishigatsubo Y Mizuki N Tugal-Tutkun I Seyahi E. Genome-Wide Association Analysis Identifies New Susceptibility Loci for Behcet’s Disease and Epistasis Between HLA-B*51 and ERAP1. Nat Genet (2013) 45(2):202–7. doi: 10.1038/ng.2520 PMC3810947 23291587

[28] Kirino Y Zhou Q Ishigatsubo Y Mizuki N Tugal-Tutkun I Seyahi E. Targeted Resequencing Implicates the Familial Mediterranean Fever Gene MEFV and the Toll-Like Receptor 4 Gene TLR4 in Behcet Disease. Proc Natl Acad Sci USA (2013) 110(20):8134–9. doi: 10.1073/pnas.1306352110 PMC3657824 23633568

[29] Montes-Cano MA Conde-Jaldon M Garcia-Lozano JR Ortiz-Fernandez L Ortego-Centeno N Castillo-Palma MJ. HLA and Non-HLA Genes in Behcet’s Disease: A Multicentric Study in the Spanish Population. Arthritis Res Ther (2013) 15(5):R145. doi: 10.1186/ar4328 24286189 PMC3978908

[30] Wu Z Zheng W Xu J Sun F Chen H Li P. IL10 Polymorphisms Associated With Behcet’s Disease in Chinese Han. Hum Immunol (2014) 75(3):271–6. doi: 10.1016/j.humimm.2013.11.009 24269690

[31] Xiang Q Chen L Hou S Fang J Zhou Y Bai L. TRAF5 and TRAF3IP2 Gene Polymorphisms are Associated With Behcet’s Disease and Vogt-Koyanagi-Harada Syndrome: A Case-Control Study. PloS One (2014) 9(1):e84214. doi: 10.1371/journal.pone.0084214 24416204 PMC3885545

[32] Ortiz-Fernandez L Garcia-Lozano JR Montes-Cano MA Conde-Jaldon M Ortego-Centeno N Garcia-Hernandez FJ. Variants of the IFI16 Gene Affecting the Levels of Expression of mRNA are Associated With Susceptibility to Behcet Disease. J Rheumatol (2015) 42(4):695–701. doi: 10.3899/jrheum.140949 25641891

[33] Sousa I Shahram F Francisco D Davatchi F Abdollahi BS Ghaderibarmi F. Brief Report: Association of CCR1, KLRC4, IL12A-AS1, STAT4, and ERAP1 With Behcet’s Disease in Iranians. Arthritis Rheumatol (2015) 67(10):2742–8. doi: 10.1002/art.39240 26097239

[34] Carapito R Shahram F Michel S Le Gentil M Radosavljevic M Meguro A. On the Genetics of the Silk Route: Association Analysis of HLA, IL10, and IL23R-IL12RB2 Regions With Behcet’s Disease in an Iranian Population. Immunogenetics (2015) 67(5-6):289–93. doi: 10.1007/s00251-015-0841-6 25940109

[35] Kappen JH Medina-Gomez C van Hagen PM Stolk L Estrada K Rivadeneira F. Genome-Wide Association Study in an Admixed Case Series Reveals IL12A as a New Candidate in Behcet Disease. PloS One (2015) 10(3):e0119085. doi: 10.1371/journal.pone.0119085 25799145 PMC4370488

[36] Xavier JM Shahram F Sousa I Davatchi F Matos M Abdollahi BS. FUT2: Filling the Gap Between Genes and Environment in Behcet’s Disease? Ann Rheum Dis (2015) 74(3):618–24. doi: 10.1136/annrheumdis-2013-204475 24326010

[37] Al-Okaily F Al-Rashidi S Al-Balawi M Mustafa M Arfin M Al-Asmari A. Genetic Association of HLA-A*26, -A*31, and -B*51 With Behcet’s Disease in Saudi Patients. Clin Med Insights Arthritis Musculoskelet Disord (2016) 9:167–73. doi: 10.4137/CMAMD.S39879 PMC4978194 27547040

[38] Lu S Song S Hou S Li H Yang P. Association of TNFSF4 Polymorphisms With Vogt-Koyanagi-Harada and Behcet’s Disease in Han Chinese. Sci Rep (2016) 6:37257. doi: 10.1038/srep37257 27872495 PMC5181833

[39] Tan H Deng B Yu H Yang Y Ding L Zhang Q. Genetic Analysis of Innate Immunity in Behcet’s Disease Identifies an Association With IL-37 and IL-18rap. Sci Rep (2016) 6:35802. doi: 10.1038/srep35802 27775096 PMC5075872

[40] Li L Yu H Jiang Y Deng B Bai L Kijlstra A. Genetic Variations of NLR Family Genes in Behcet’s Disease. Sci Rep (2016) 6:20098. doi: 10.1038/srep20098 26833430 PMC4735577

[41] Ortiz-Fernandez L Carmona FD Montes-Cano MA Garcia-Lozano JR Conde-Jaldon M Ortego-Centeno N. Genetic Analysis With the Immunochip Platform in Behcet Disease. Identification of Residues Associated in the HLA Class I Region and New Susceptibility Loci. PloS One (2016) 11(8):e0161305. doi: 10.1371/journal.pone.0161305 27548383 PMC4993481

[42] Qin X Xu J Wu Z Sun F Chen H Zheng W. Association Study of Rs924080 and Rs11209032 Polymorphisms of IL23R-IL12RB2 in a Northern Chinese Han Population With Behcet’s Disease. Hum Immunol (2016) 77(12):1284–90. doi: 10.1016/j.humimm.2016.09.006 27660093

[43] Yu H Zheng M Zhang L Li H Zhu Y Cheng L. Identification of Susceptibility SNPs in IL10 and IL23R-IL12RB2 for Behcet’s Disease in Han Chinese. J Allergy Clin Immunol (2017) 139(2):621–7. doi: 10.1016/j.jaci.2016.05.024 27464962

[44] Takeuchi M Mizuki N Meguro A Ombrello MJ Kirino Y Satorius C. Dense Genotyping of Immune-Related Loci Implicates Host Responses to Microbial Exposure in Behcet’s Disease Susceptibility. Nat Genet (2017) 49(3):438–43. doi: 10.1038/ng.3786 PMC5369770 28166214

[45] Kang EH Kim S Park MY Choi JY Choi IA Kim MJ. Behcet’s Disease Risk Association Fine-Mapped on the IL23R-IL12RB2 Intergenic Region in Koreans. Arthritis Res Ther (2017) 19(1):227. doi: 10.1186/s13075-017-1435-5 29017598 PMC5633897

[46] Jiang Y Cheng L Li X Zhou W Zhang L. Associations Between TNFSF4, TNFSF8 and TNFSF15 and Behcet’s Disease But Not VKH Syndrome in Han Chinese. Oncotarget. (2017) 8(62):105037–46. doi: 10.18632/oncotarget.22064 PMC5739618 29285231

[47] Wu P Du L Hou S Su G Yang L Hu J. Association of LACC1, CEBPB-PTPN1, RIPK2 and ADO-EGR2 With Ocular Behcet’s Disease in a Chinese Han Population. Br J Ophthalmol (2018) 102(9):1308–14. doi: 10.1136/bjophthalmol-2017-311753 PMC6104672 29907633

[48] Zhang D Qin J Li L Su G Huang G Cao Q. Analysis of the Association Between Fc Receptor Family Gene Polymorphisms and Ocular Behcet’s Disease in Han Chinese. Sci Rep (2018) 8(1):4850. doi: 10.1038/s41598-018-23222-8 29555961 PMC5859267

[49] Tan X Zhou Q Lv M Tan H Wang Q Zhang L. Functional Genetic Polymorphisms in the IL1RL1-IL18R1 Region Confer Risk for Ocular Behcet’s Disease in a Chinese Han Population. Front Genet (2020) 11:645. doi: 10.3389/fgene.2020.00645 32719716 PMC7350896

[50] Gholijani N Daryabor G Kalantar K Yazdani MR Shenavandeh S Zahed M. Interleukin-27 Gene Variant Rs153109 is Associated With Enhanced Cytokine Serum Levels and Susceptibility to Behcet’s Disease in the Iranian Population. Eur Cytokine Netw (2020) 31(4):140–6. doi: 10.1684/ecn.2020.0458 33648922

[51] Padula MC Leccese P Lascaro N Radice RP Limongi AR Sorrento GG. Correlation of Tumor Necrosis Factor-Alpha -308G>A Polymorphism With Susceptibility, Clinical Manifestations, and Severity in Behcet Syndrome: Evidences From an Italian Genetic Case-Control Study. DNA Cell Biol (2020) 39(7):1104–10. doi: 10.1089/dna.2020.5361 32352842

[52] Su G Zhong Z Zhou Q Du L Ye Z Li F. Identification of Novel Risk Loci for Behcet’s Disease-Related Uveitis in a Chinese Population in a Genome-Wide Association Study. Arthritis Rheumatol (2022) 74(4):671–81. doi: 10.1002/art.41998 34652073

[53] Feng M Zhou S Liu T Yu Y Su Q Li X. Association Between Interleukin 35 Gene Single Nucleotide Polymorphisms and the Uveitis Immune Status in a Chinese Han Population. Front Immunol (2021) 12:758554. doi: 10.3389/fimmu.2021.758554 34950136 PMC8688856

[54] Ortiz Fernandez L Coit P Yilmaz V Yentur SP Alibaz-Oner F Aksu K. Genetic Association of a Gain-Of-Function IFNGR1 Polymorphism and the Intergenic Region LNCAROD/DKK1 With Behcet’s Disease. Arthritis Rheumatol (2021) 73(7):1244–52. doi: 10.1002/art.41637 PMC8238846 33393726

[55] Kamal A Elgengehy FT Elawady Z Fawzy NA El Sisi O. Role of miR-146a Rs2910164 and UTS2 Rs228648 Genetic Variants in Behcet’s Disease. Immunol Invest. (2021) 19: 1–10. doi: 10.1080/08820139.2021.1883647 33605830

[56] Hou S Qi J Liao D Zhang Q Fang J Zhou Y. Copy Number Variations of Complement Component C4 are Associated With Behcet’s Disease But Not With Ankylosing Spondylitis Associated With Acute Anterior Uveitis. Arthritis Rheumatol (2013) 65(11):2963–70. doi: 10.1002/art.38116 23918728

[57] Fang J Chen L Tang J Hou S Liao D Ye Z. Association Between Copy Number Variations of TLR7 and Ocular Behcet’s Disease in a Chinese Han Population. Invest Ophthalmol Vis Sci (2015) 56(3):1517–23. doi: 10.1167/iovs.14-15030 25650422

[58] Xu D Hou S Zhang J Jiang Y Kijlstra A Yang P. Copy Number Variations and Gene Polymorphisms of Complement Components in Ocular Behcet’s Disease and Vogt-Koyanagi-Harada Syndrome. Sci Rep (2015) 5:12989. doi: 10.1038/srep12989 26269006 PMC4534762

[59] Hou S Liao D Zhang J Fang J Chen L Qi J. Genetic Variations of IL17F and IL23A Show Associations With Behcet’s Disease and Vogt-Koyanagi-Harada Syndrome. Ophthalmology. (2015) 122(3):518–23. doi: 10.1016/j.ophtha.2014.09.025 25439430

[60] Liao D Hou S Zhang J Fang J Liu Y Bai L. Copy Number Variants and Genetic Polymorphisms in TBX21, GATA3, Rorc, Foxp3 and Susceptibility to Behcet’s Disease and Vogt-Koyanagi-Harada Syndrome. Sci Rep (2015) 5:9511. doi: 10.1038/srep09511 25873156 PMC4397537

[61] Yu H Luo L Wu L Zheng M Zhang L Liu Y. FAS Gene Copy Numbers are Associated With Susceptibility to Behcet Disease and VKH Syndrome in Han Chinese. Hum Mutat (2015) 36(11):1064–9. doi: 10.1002/humu.22829 26136352

[62] Ma X Wang X Zheng G Tan G Zhou F Wei W. Critical Role of Gut Microbiota and Epigenetic Factors in the Pathogenesis of Behcet’s Disease. Front Cell Dev Biol (2021) 9:719235. doi: 10.3389/fcell.2021.719235 34676209 PMC8525702

[63] Mizuki N Ota M Kimura M Ohno S Ando H Katsuyama Y. Triplet Repeat Polymorphism in the Transmembrane Region of the MICA Gene: A Strong Association of Six GCT Repetitions With Behcet Disease. Proc Natl Acad Sci USA (1997) 94(4):1298–303. doi: 10.1073/pnas.94.4.1298 PMC19785 9037047

[64] Deng Y Zhu W Zhou X. Immune Regulatory Genes Are Major Genetic Factors to Behcet Disease: Systematic Review. Open Rheumatol J (2018) 12:70–85. doi: 10.2174/1874312901812010070 30069262 PMC6040213

[65] Hughes T Coit P Adler A Yilmaz V Aksu K Duzgun N. Identification of Multiple Independent Susceptibility Loci in the HLA Region in Behcet’s Disease. Nat Genet (2013) 45(3):319–24. doi: 10.1038/ng.2551 23396137

[66] Yazici H Chamberlain MA Schreuder I D’Amaro J Muftuoglu M. HLA Antigens in Behcet’s Disease: A Reappraisal by a Comparative Study of Turkish and British Patients. Ann Rheum Dis (1980) 39(4):344–8. doi: 10.1136/ard.39.4.344 PMC1000554 7436560

[67] Gul A Ohno S. HLA-B*51 and Behcet Disease. Ocul Immunol Inflamm (2012) 20(1):37–43. doi: 10.3109/09273948.2011.634978 22188278

[68] Horie Y Meguro A Ohta T Lee EB Namba K Mizuuchi K. HLA-B51 Carriers are Susceptible to Ocular Symptoms of Behcet Disease and the Association Between the Two Becomes Stronger Towards the East Along the Silk Road: A Literature Survey. Ocul Immunol Inflamm (2017) 25(1):37–40. doi: 10.3109/09273948.2015.1136422 26954704

[69] de Menthon M Lavalley MP Maldini C Guillevin L Mahr A. HLA-B51/B5 and the Risk of Behcet’s Disease: A Systematic Review and Meta-Analysis of Case-Control Genetic Association Studies. Arthritis Rheumatol (2009) 61(10):1287–96. doi: 10.1002/art.24642 PMC3867978 19790126

[70] Leon Machado JA Steimle V. The MHC Class II Transactivator CIITA: Not (Quite) the Odd-One-Out Anymore Among NLR Proteins. Int J Mol Sci (2021) 22(3):1074. doi: 10.3390/ijms22031074 33499042 PMC7866136

[71] Zhang J Liao D Yang L Hou S. Association Between Functional MICA-TM and Behcet’s Disease: A Systematic Review and Meta-Analysis. Sci Rep (2016) 6:21033. doi: 10.1038/srep21033 26875668 PMC4753467

[72] Groh V Steinle A Bauer S Spies T. Recognition of Stress-Induced MHC Molecules by Intestinal Epithelial Gammadelta T Cells. Science. (1998) 279(5357):1737–40. doi: 10.1126/science.279.5357.1737 9497295

[73] Wei F Zhang YU Li W. A Meta-Analysis of the Association Between Behcet’s Disease and MICA-A6. BioMed Rep (2016) 4(6):741–5. doi: 10.3892/br.2016.644 PMC4887777 27284416

[74] Fiorentino DF Zlotnik A Vieira P Mosmann TR Howard M Moore KW. IL-10 Acts on the Antigen-Presenting Cell to Inhibit Cytokine Production by Th1 Cells. J Immunol (1991) 146(10):3444–51. 1827484

[75] Zhu W Deng Y Wang J Guo X Ding W Chao J. MICA*049, Not MICA*009, is Associated With Behcet’s Disease in a Chinese Population. Sci Rep (2019) 9(1):10856. doi: 10.1038/s41598-019-47289-z 31350414 PMC6659628

[76] Shimizu J Takai K Fujiwara N Arimitsu N Ueda Y Wakisaka S. Excessive CD4+ T Cells Co-Expressing Interleukin-17 and Interferon-Gamma in Patients With Behcet’s Disease. Clin Exp Immunol (2012) 168(1):68–74. doi: 10.1111/j.1365-2249.2011.04543.x 22385240 PMC3390496

[77] Zhong Z Su G Kijlstra A Yang P. Activation of the Interleukin-23/Interleukin-17 Signalling Pathway in Autoinflammatory and Autoimmune Uveitis. Prog Retin Eye Res (2021) 80:100866. doi: 10.1016/j.preteyeres.2020.100866 32422390

[78] Vural S Kerl K Ertop Dogan P Vollmer S Puchta U He M. Lesional Activation of Tc 17 Cells in Behcet Disease and Psoriasis Supports HLA Class I-Mediated Autoimmune Responses. Br J Dermatol (2021) 185(6):1209–20. doi: 10.1111/bjd.20643 34254298

[79] Pay S Simsek I Erdem H Pekel A Musabak U Sengul A. Dendritic Cell Subsets and Type I Interferon System in Behcet’s Disease: Does Functional Abnormality in Plasmacytoid Dendritic Cells Contribute to Th1 Polarization? Clin Exp Rheumatol (2007) 25(4 Suppl 45):S34–40. 17949549

[80] Hirahara L Takase-Minegishi K Kirino Y Iizuka-Iribe Y Soejima Y Yoshimi R. The Roles of Monocytes and Macrophages in Behcet’s Disease With Focus on M1 and M2 Polarization. Front Immunol (2022) 13:852297. doi: 10.3389/fimmu.2022.852297 35359926 PMC8963421

[81] Petrushkin H Hasan MS Stanford MR Fortune F Wallace GR. Behcet’s Disease: Do Natural Killer Cells Play a Significant Role? Front Immunol (2015) 6:134. doi: 10.3389/fimmu.2015.00134 25852697 PMC4371743

[82] Emmi G Becatti M Bettiol A Hatemi G Prisco D Fiorillo C. Behcet’s Syndrome as a Model of Thrombo-Inflammation: The Role of Neutrophils. Front Immunol (2019) 10:1085. doi: 10.3389/fimmu.2019.01085 31139195 PMC6527740

[83] Suzuki N Sakane T Ueda Y Tsunematsu T. Abnormal B Cell Function in Patients With Behcet’s Disease. Arthritis Rheumatol (1986) 29(2):212–9. doi: 10.1002/art.1780290209 3485432

[84] Hetta HF Mohamed AAA Zahran AM AM S My Sayed M Ga Saleh M. Possible Role of Regulatory B Cells in Different Behcet’s Disease Phenotypes and Therapies: First Report From Egypt. J Inflammation Res (2021) 14:737–44. doi: 10.2147/JIR.S279912 PMC7955029 33727848

[85] Saraiva M O’Garra A. The Regulation of IL-10 Production by Immune Cells. Nat Rev Immunol (2010) 10(3):170–81. doi: 10.1038/nri2711 20154735

[86] Vivas MC Villamarin-Guerrero HF Sanchez CA. Interleukin-10 (IL-10) 1082 Promoter Polymorphisms and Plasma IL-10 Levels in Patients With Bacterial Sepsis. Rom J Intern Med (2021) 59(1):50–7. doi: 10.2478/rjim-2020-0033 33155998

[87] Barrett JC Clayton DG Concannon P Akolkar B Cooper JD Erlich HA. Genome-Wide Association Study and Meta-Analysis Find That Over 40 Loci Affect Risk of Type 1 Diabetes. Nat Genet (2009) 41(6):703–7. doi: 10.1038/ng.381 PMC2889014 19430480

[88] Paradowska-Gorycka A Jurkowska M Czuszynska Z Felis-Giemza A Manczak M Zdrojewski Z. IL-10, IL-12B and IL-17 Gene Polymorphisms in Patients With Mixed Connective Tissue Disease. Mod Rheumatol (2015) 25(3):487–9. doi: 10.3109/14397595.2014.951143 25159155

[89] Braga M Lara-Armi FF Neves JSF Rocha-Loures MA Terron-Monich MS Bahls-Pinto LD. Influence of IL10 (Rs1800896) Polymorphism and TNF-Alpha, IL-10, IL-17A, and IL-17f Serum Levels in Ankylosing Spondylitis. Front Immunol (2021) 12:653611. doi: 10.3389/fimmu.2021.653611 34290697 PMC8287882

[90] Wallace GR Kondeatis E Vaughan RW Verity DH Chen Y Fortune F. IL-10 Genotype Analysis in Patients With Behcet’s Disease. Hum Immunol (2007) 68(2):122–7. doi: 10.1016/j.humimm.2006.11.010 17321902

[91] Nakano H Kirino Y Takeno M Higashitani K Nagai H Yoshimi R. GWAS-Identified CCR1 and IL10 Loci Contribute to M1 Macrophage-Predominant Inflammation in Behcet’s Disease. Arthritis Res Ther (2018) 20(1):124. doi: 10.1186/s13075-018-1613-0 29895319 PMC5998575

[92] Tan H Su G Tan X Qin Y Chen L Yuan G. SNP-Mediated Binding of TBX1 to the Enhancer Element of IL-10 Reduces the Risk of Behcet’s Disease. Epigenomics. (2021) 13(19):1523–37. doi: 10.2217/epi-2021-0215 34612069

[93] Murphy CA Langrish CL Chen Y Blumenschein W McClanahan T Kastelein RA. Divergent Pro- and Antiinflammatory Roles for IL-23 and IL-12 in Joint Autoimmune Inflammation. J Exp Med (2003) 198(12):1951–7. doi: 10.1084/jem.20030896 PMC2194162 14662908

[94] Iwakura Y Ishigame H. The IL-23/IL-17 Axis in Inflammation. J Clin Invest. (2006) 116(5):1218–22. doi: 10.1172/JCI28508 PMC1451213 16670765

[95] Khaib Dit Naib O Aribi M Idder A Chiali A Sairi H Touitou I. Association Analysis of IL10, TNF-Alpha, and IL23R-IL12RB2 SNPs With Behcet’s Disease Risk in Western Algeria. Front Immunol (2013) 4:342. doi: 10.3389/fimmu.2013.00342 24151497 PMC3801160

[96] Blumberg H Dinh H Dean C Jr. Trueblood ES Bailey K Shows D. IL-1RL2 and its Ligands Contribute to the Cytokine Network in Psoriasis. J Immunol (2010) 185(7):4354–62. doi: 10.4049/jimmunol.1000313 20833839

[97] Pastorelli L Garg RR Hoang SB Spina L Mattioli B Scarpa M. Epithelial-Derived IL-33 and its Receptor ST2 are Dysregulated in Ulcerative Colitis and in Experimental Th1/Th2 Driven Enteritis. Proc Natl Acad Sci USA (2010) 107(17):8017–22. doi: 10.1073/pnas.0912678107 PMC2867895 20385815

[98] Gutcher I Urich E Wolter K Prinz M Becher B. Interleukin 18-Independent Engagement of Interleukin 18 Receptor-Alpha is Required for Autoimmune Inflammation. Nat Immunol (2006) 7(9):946–53. doi: 10.1038/ni1377 16906165

[99] Vonk JM Nieuwenhuis MAE Dijk FN Boudier A Siroux V Bouzigon E. Novel Genes and Insights in Complete Asthma Remission: A Genome-Wide Association Study on Clinical and Complete Asthma Remission. Clin Exp Allergy (2018) 48(10):1286–96. doi: 10.1111/cea.13181 29786918

[100] Hirota T Takahashi A Kubo M Tsunoda T Tomita K Sakashita M. Genome-Wide Association Study Identifies Eight New Susceptibility Loci for Atopic Dermatitis in the Japanese Population. Nat Genet (2012) 44(11):1222–6. doi: 10.1038/ng.2438 23042114

[101] Kinoshita K Yamagata T Nozaki Y Sugiyama M Ikoma S Funauchi M. Blockade of IL-18 Receptor Signaling Delays the Onset of Autoimmune Disease in MRL-Faslpr Mice. J Immunol (2004) 173(8):5312–8. doi: 10.4049/jimmunol.173.8.5312 15470078

[102] Alipour S Nouri M Sakhinia E Samadi N Roshanravan N Ghavami A. Epigenetic Alterations in Chronic Disease Focusing on Behcet’s Disease: Review. BioMed Pharmacother. (2017) 91:526–33. doi: 10.1016/j.biopha.2017.04.106 28482290

[103] Akbaba TH Sag E Balci-Peynircioglu B Ozen S. Epigenetics for Clinicians From the Perspective of Pediatric Rheumatic Diseases. Curr Rheumatol Rep (2020) 22(8):46. doi: 10.1007/s11926-020-00912-9 32654092

[104] Kolahi S Farajzadeh MJ Alipour S Abhari A Farhadi J Bahavarnia N. Determination of Mir-155 and Mir-146a Expression Rates and its Association With Expression Level of TNF-Alpha and CTLA4 Genes in Patients With Behcet’s Disease. Immunol Lett (2018) 204:55–9. doi: 10.1016/j.imlet.2018.10.012 30366049

[105] Jadideslam G Ansarin K Sakhinia E Babaloo Z Abhari A Alipour S. Expression Levels of miR-21, miR-146b and miR-326 as Potential Biomarkers in Behcet’s Disease. biomark Med (2019) 13(16):1339–48. doi: 10.2217/bmm-2019-0098 31599663

[106] Yu H Liu Y Bai L Kijlstra A Yang P. Predisposition to Behcet’s Disease and VKH Syndrome by Genetic Variants of miR-182. J Mol Med (Berl). (2014) 92(9):961–7. doi: 10.1007/s00109-014-1159-9 24801147

[107] Woo MY Yun SJ Cho O Kim K Lee ES Park S. MicroRNAs Differentially Expressed in Behcet Disease are Involved in Interleukin-6 Production. J Inflammation (Lond). (2016) 13:22. doi: 10.1186/s12950-016-0130-7 PMC4952146 27441030

[108] Karasneh J Gul A Ollier WE Silman AJ Worthington J. Whole-Genome Screening for Susceptibility Genes in Multicase Families With Behcet’s Disease. Arthritis Rheumatol (2005) 52(6):1836–42. doi: 10.1002/art.21060 15934084

[109] Ahmadi M Yousefi M Abbaspour-Aghdam S Dolati S Aghebati-Maleki L Eghbal-Fard S. Disturbed Th17/Treg Balance, Cytokines, and miRNAs in Peripheral Blood of Patients With Behcet’s Disease. J Cell Physiol (2019) 234(4):3985–94. doi: 10.1002/jcp.27207 30317557

[110] Aletaha D Smolen JS. Diagnosis and Management of Rheumatoid Arthritis: A Review. JAMA. (2018) 320(13):1360–72. doi: 10.1001/jama.2018.13103 30285183

[111] Mease P Hall S FitzGerald O van der Heijde D Merola JF Avila-Zapata F. Tofacitinib or Adalimumab Versus Placebo for Psoriatic Arthritis. N Engl J Med (2017) 377(16):1537–50. doi: 10.1056/NEJMoa1615975 29045212

[112] Sandborn WJ Ghosh S Panes J Vranic I Su C Rousell S. Tofacitinib, an Oral Janus Kinase Inhibitor, in Active Ulcerative Colitis. N Engl J Med (2012) 367(7):616–24. doi: 10.1056/NEJMoa1112168 22894574

[113] Papp KA Menter MA Abe M Elewski B Feldman SR Gottlieb AB. Tofacitinib, an Oral Janus Kinase Inhibitor, for the Treatment of Chronic Plaque Psoriasis: Results From Two Randomized, Placebo-Controlled, Phase III Trials. Br J Dermatol (2015) 173(4):949–61. doi: 10.1111/bjd.14018 26149717

[114] Paley MA Karacal H Rao PK Margolis TP Miner JJ. Tofacitinib for Refractory Uveitis and Scleritis. Am J Ophthalmol Case Rep (2019) 13:53–5. doi: 10.1016/j.ajoc.2018.12.001 PMC6288302 30582071

[115] Bauermann P Heiligenhaus A Heinz C. Effect of Janus Kinase Inhibitor Treatment on Anterior Uveitis and Associated Macular Edema in an Adult Patient With Juvenile Idiopathic Arthritis. Ocul Immunol Inflamm (2019) 27(8):1232–4. doi: 10.1080/09273948.2019.1605453 31268748

[116] Pyare R Kaushik V Dutta Majumder P Biswas J. Tofacitinib in Recalcitrant Scleritis: First Case Report From India. Indian J Ophthalmol (2020) 68(9):1988–90. doi: 10.4103/ijo.IJO_534_20 PMC7690527 32823452

[117] Liu J Hou Y Sun L Li C Li L Zhao Y. A Pilot Study of Tofacitinib for Refractory Behcet’s Syndrome. Ann Rheum Dis (2020) 79(11):1517–20. doi: 10.1136/annrheumdis-2020-217307 32461206

[118] Hou S Qi J Zhang Q Liao D Li Q Hu K. Genetic Variants in the JAK1 Gene Confer Higher Risk of Behcet’s Disease With Ocular Involvement in Han Chinese. Hum Genet (2013) 132(9):1049–58. doi: 10.1007/s00439-013-1312-5 23674219

[119] Yang C Mai H Peng J Zhou B Hou J Jiang D. STAT4: An Immunoregulator Contributing to Diverse Human Diseases. Int J Biol Sci (2020) 16(9):1575–85. doi: 10.7150/ijbs.41852 PMC7097918 32226303

[120] Korman BD Kastner DL Gregersen PK Remmers EF. STAT4: Genetics, Mechanisms, and Implications for Autoimmunity. Curr Allergy Asthma Rep (2008) 8(5):398–403. doi: 10.1007/s11882-008-0077-8 18682104 PMC2562257

[121] Remmers EF Plenge RM Lee AT Graham RR Hom G Behrens TW. STAT4 and the Risk of Rheumatoid Arthritis and Systemic Lupus Erythematosus. N Engl J Med (2007) 357(10):977–86. doi: 10.1056/NEJMoa073003 PMC2630215 17804842

[122] Ebrahimiyan H Mostafaei S Aslani S Jamshidi A Mahmoudi M. Studying the Association Between STAT4 Gene Polymorphism and Susceptibility to Rheumatoid Arthritis Disease: An Updated Meta-Analysis. Iran J Immunol (2019) 16(1):71–83. doi: 10.1016/j.mgene.2018.03.010 30864557

[123] Potter C Eyre S Cope A Worthington J Barton A. Investigation of Association Between the TRAF Family Genes and RA Susceptibility. Ann Rheum Dis (2007) 66(10):1322–6. doi: 10.1136/ard.2006.065706 PMC1994286 17277003

[124] Wang Q Yi S Du Z Huang X Xu J Cao Q. The Rs12569232 SNP Association With Vogt-Koyanagi-Harada Disease and Behcet’s Disease Is Probably Mediated by Regulation of Linc00467 Expression. Ocul Immunol Inflamm (2021) 29(7-8):1464–70. doi: 10.1080/09273948.2020.1745244 32400232

[125] Yalcin B Atakan N Dogan S. Association of Interleukin-23 Receptor Gene Polymorphism With Behcet Disease. Clin Exp Dermatol (2014) 39(8):881–7. doi: 10.1111/ced.12400 25156021

[126] Arslan Tas D Erken E Yildiz F Dinkci S Sakalli H. Mevalonate Kinase Gene Mutations and Their Clinical Correlations in Behcet’s Disease. Int J Rheum Dis (2014) 17(4):435–43. doi: 10.1111/1756-185X.12243 24411001

[127] Mahmoudi M Ashraf-Ganjouei A Javinani A Shahram F Meguro A Mizuki N. Epistatic Interaction of ERAP1 and HLA-B*51 in Iranian Patients With Behcet’s Disease. Sci Rep (2018) 8(1):17612. doi: 10.1038/s41598-018-35700-0 30514861 PMC6279803

[128] Ozguclu S Duman T Ates FSO Kucuksahin O Colak S Olmez U. Serum Interleukin-37 Level and Interleukin-37 Gene Polymorphism in Patients With Behcet Disease. Clin Rheumatol (2019) 38(2):495–502. doi: 10.1007/s10067-018-4288-7 30225559

[129] Inal EE Rustemoglu A Inanir A Ekinci D Gul U Yigit S. Associations of Rs4810485 and Rs1883832 Polymorphisms of CD40 Gene With Susceptibility and Clinical Findings of Behcet’s Disease. Rheumatol Int (2015) 35(5):837–43. doi: 10.1007/s00296-014-3171-3 25373542

[130] Talaat RM Ashour ME Bassyouni IH Raouf AA. Polymorphisms of Interleukin 6 and Interleukin 10 in Egyptian People With Behcet’s Disease. Immunobiol (2014) 219(8):573–82. doi: 10.1016/j.imbio.2014.03.004 24703990

[131] Kim ES Kim SW Moon CM Park JJ Kim TI Kim WH. Interactions Between IL17A, IL23R, and STAT4 Polymorphisms Confer Susceptibility to Intestinal Behcet’s Disease in Korean Population. Life Sci (2012) 90(19-20):740–6. doi: 10.1016/j.lfs.2012.03.017 22483685

[132] Abdel Galil SM Hagrass HA. The Role of CTLA-4 Exon-1 49 a/G Polymorphism and Soluble CTLA-4 Protein Level in Egyptian Patients With Behcet’s Disease. BioMed Res Int (2014) 2014:513915. doi: 10.1155/2014/513915 24991555 PMC4060294

[133] Wu Z Chen H Sun F Xu J Zheng W Li P. PTPN2 Rs1893217 Single-Nucleotide Polymorphism is Associated With Risk of Behcet’s Disease in a Chinese Han Population. Clin Exp Rheumatol (2014) 32(4 Suppl 84):S20–6. 24480412

[134] Zheng X Wang D Hou S Zhang C Lei B Xiao X. Association of Macrophage Migration Inhibitory Factor Gene Polymorphisms With Behcet’s Disease in a Han Chinese Population. Ophthalmol (2012) 119(12):2514–8. doi: 10.1016/j.ophtha.2012.06.039 22939113

[135] Hu J Hou S Zhu X Fang J Zhou Y Liu Y. Interleukin-10 Gene Polymorphisms are Associated With Behcet’s Disease But Not With Vogt-Koyanagi-Harada Syndrome in the Chinese Han Population. Mol Vis (2015) 21:589–603. 26015771 PMC4443582

[136] Padula MC Leccese P Lascaro N Padula AA Carbone T Martelli G. A First Step for the Molecular Characterization of Neurological Involvement of Behcet Syndrome: An Italian Pivotal Study. J Mol Neurosci (2021) 71(6):1284–9. doi: 10.1007/s12031-020-01755-w 33216288

[137] Mace A Kutalik Z Valsesia A. Copy Number Variation. Methods Mol Biol (2018) 1793:231–58. doi: 10.1007/978-1-4939-7868-7_14 29876900

[138] Boteva L Morris DL Cortes-Hernandez J Martin J Vyse TJ Fernando MM. Genetically Determined Partial Complement C4 Deficiency States are Not Independent Risk Factors for SLE in UK and Spanish Populations. Am J Hum Genet (2012) 90(3):445–56. doi: 10.1016/j.ajhg.2012.01.012 PMC3309188 22387014

[139] Rigby WF Wu YL Zan M Zhou B Rosengren S Carlson C. Increased Frequency of Complement C4B Deficiency in Rheumatoid Arthritis. Arthritis Rheumatol (2012) 64(5):1338–44. doi: 10.1002/art.33472 PMC3775471 22076784

[140] Liu YH Wan L Chang CT Liao WL Chen WC Tsai Y. Association Between Copy Number Variation of Complement Component C4 and Graves’ Disease. J BioMed Sci (2011) 18:71. doi: 10.1186/1423-0127-18-71 21943165 PMC3212822

[141] Chang JH McCluskey P Wakefield D. Expression of Toll-Like Receptor 4 and its Associated Lipopolysaccharide Receptor Complex by Resident Antigen-Presenting Cells in the Human Uvea. Invest Ophthalmol Vis Sci (2004) 45(6):1871–8. doi: 10.1167/iovs.03-1113 15161852

[142] Ortiz-Fernandez L Garcia-Lozano JR Montes-Cano MA Conde-Jaldon M Leo E Ortego-Centeno N. Association of Haplotypes of the TLR8 Locus With Susceptibility to Crohn’s and Behcet’s Diseases. Clin Exp Rheumatol (2015) 33(6 Suppl 94):S117–22. 26486764

[143] Wajant H. The Fas Signaling Pathway: More Than a Paradigm. Science. (2002) 296(5573):1635–6. doi: 10.1126/science.1071553 12040174

[144] Fan X Shangguan L Li M Li CY Liu B. Functional Polymorphisms of the FAS/FASLG Genes are Associated With Risk of Alopecia Areata in a Chinese Population: A Case-Control Analysis. Br J Dermatol (2010) 163(2):340–4. doi: 10.1111/j.1365-2133.2010.09808.x 20394629

[145] Yildir S Sezgin M Barlas IO Turkoz G Ankarali HC Sahin G. Relation of the Fas and FasL Gene Polymorphisms With Susceptibility to and Severity of Rheumatoid Arthritis. Rheumatol Int (2013) 33(10):2637–45. doi: 10.1007/s00296-013-2793-1 23749041

[146] Allis CD Jenuwein T. The Molecular Hallmarks of Epigenetic Control. Nat Rev Genet (2016) 17(8):487–500. doi: 10.1038/nrg.2016.59 27346641

[147] Alipour S Sakhinia E Khabbazi A Samadi N Babaloo Z Azad M. Methylation Status of Interleukin-6 Gene Promoter in Patients With Behcet’s Disease. Reumatol Clin (Engl Ed) (2020) 16(3):229–34. doi: 10.1016/j.reuma.2018.06.006 30076035

[148] Alipour S Nouri M Khabbazi A Samadi N Babaloo Z Abolhasani S. Hypermethylation of IL-10 Gene Is Responsible for its Low mRNA Expression in Behcet’s Disease. J Cell Biochem (2018) 119(8):6614–22. doi: 10.1002/jcb.26809 29719061

[149] Yu H Du L Yi S Wang Q Zhu Y Qiu Y. Epigenome-Wide Association Study Identifies Behcet’s Disease-Associated Methylation Loci in Han Chinese. Rheumatol (Oxford). (2019) 58(9):1574–84. doi: 10.1093/rheumatology/kez043 30863869

[150] Gardner PJ Joshi L Lee RW Dick AD Adamson P Calder VL. SIRT1 Activation Protects Against Autoimmune T Cell-Driven Retinal Disease in Mice *via* Inhibition of IL-2/Stat5 Signaling. J Autoimmun (2013) 42:117–29. doi: 10.1016/j.jaut.2013.01.011 23395551

